# Remembering the Children: Implementation and Success of a Robust Method for Identifying and Testing Children of HIV Patients

**DOI:** 10.1155/2013/828024

**Published:** 2013-10-30

**Authors:** Christopher Darlow, Peter Tovey, Fiona Wallis, Clare Knowles, Ian Fairley, Charles Lacey, Fabiola Martin

**Affiliations:** ^1^York Teaching Hospital NHS Trust, Mongate Health Center, 31 Monkgate, York YO31 7WA, UK; ^2^Centre for Immunology and Infection, Department of Biology, HYMS, University of York, Heslington, York YO10 5DD, UK

## Abstract

*Background.* Children of HIV patients are a historically neglected demographic by HIV services. It has been recommended by CHIVA that HIV services have a robust method of detecting and testing untested children. We note that no such method is either in widespread use or in the literature. *Method.* In December 2011, a one-page proforma to identify HIV untested children and a clear multidisciplinary pathway to test them were implemented. Twelve months later the uptake of the proforma and pathway, the numbers of patients and children identified for testing, and their outcomes were audited. *Results.* The proforma was completed in 192/203 (94.6%) eligible patients. Twenty-five (21.5%) of 118 identified offspring had not been tested. Ten (8.5%) of these were <18 years old. All were reported to be clinically well. Ten children were referred for testing, seven were tested immediately, and three were tested within 18 months of identification. All children were tested HIV negative. *Discussion.* We have identified a method of identification that is easy and robust and provides a user-friendly safety net to empower healthcare providers to identify and test children at risk. We recommend the implementation of such strategies nationwide to prevent death due to undiagnosed HIV in children.

## 1. Introduction

HIV infection in children is an important public health issue. Although global transmission rates are decreasing, 3.3 million children under the age of 14 are infected with HIV of whom 330,000 contracted the virus in 2011 [1]. In contrast, vertical transmission rates are <1% in the UK due the successful UK antenatal HIV testing program and routine postdelivery HIV testing of all children born to HIV-positive mothers [2].

However, there are still children living in the UK who are HIV-positive without being tested [3] with potentially catastrophic consequences. These are mostly children of mothers who have received their antenatal care outside the UK. Children with HIV may remain asymptomatic until adolescence and early adulthood [4] and therefore present late in their disease. In the period of 2003–2006, 18 children were known to have died from HIV, nine within one month of their HIV diagnosis [5]. In order to prevent HIV associated morbidity and mortality it is important to identify children of HIV-positive patients who have never been HIV tested, regardless of age.

The children of HIV patients are a historically poorly captured demographic within UK HIV medicine services [6, 7] for a multitude of reasons.

1. Poor and inconsistent data collection on the children of HIV-positive parents;
2. Parents may be worried and feel ashamed about potential disclosure of their HIV status to their children;
3. Parents may feel anxious and guilty about having a potential HIV-positive child;
4. Parents and healthcare professionals may feel that a child, who has been well for many years, erroneously does not need testing;
5. Healthcare professionals may have apprehensions about the breakdown of a trusting relationship with their patients by broaching the HIV testing of their children.

Following the sudden AIDS related death of a 10-year-old child born to a parent who had been in HIV care for the previous six years, the Children's HIV Association (CHIVA) and the British HIV Association (BHIVA) published “Don't Forget the Children” in 2009 [8]. This document recomends the following.

1. All adult HIV services, including statutory and voluntary, as well as NHS and social services, must have protocols and procedures in place to ensure that all children of HIV-positive parents are tested for HIV.
2. A multisector, multidisciplinary team (MDT) needs to be identified for each HIV service, or network, and be responsible for setting up protocols and managing cases if and when they arise.
3. All HIV units will need to perform a “look back” exercise to establish the HIV status of any children whose HIV-positive parents attend that service.
4. All HIV services need effective operational procedures and information systems to monitor this process comprehensively.
5. All new HIV-positive patients attending adult HIV services should have all children identified and tested and the information clearly documented.
6. There needs to be joint protocols in place between health and social care to manage those cases where parents initially refuse, in order that these cases may be dealt with sensitively and appropriately. A clear pathway of referral needs to be identified within the multidisciplinary team.
7. The process of identifying children to performing an HIV test is completed within a time period of 12 months [9].

Historically, patients at our unit were questioned about living with, having children, and the children's HIV status opportunistically. We could not find a national or internationally agreed systematic method for implementing the “Don't Forget the Children” recommendations. Therefore, we developed a new strategy to identify and test the children of HIV-positive parents attending the North Yorkshire HIV services led by York Teaching Hospital. We audited its effectiveness 12 months later. The aim of this report is to introduce the strategy and to inform of the outcomes of its implementation.

## 2. Methods

The Strategy Implemented Was Three-Pronged. First, we set up a regular three-monthly MDT meeting with local paediatricians at Harrogate and York Teaching Hospital and established a social service referral pathway should a parent refuse to cooperate.

Secondly, we developed five HIV testing options offered to HIV-positive parents emphasising our aim to safeguard confidentiality. Patients could chose to have their children HIV tested by (i) general practitioner (GP), (ii) TB services as part of immigration check, (iii) paediatrician, (iv) provider referral if the child was an adult and/or sexually active, and (v) HIV specialist nurse.

**Figure 1:**
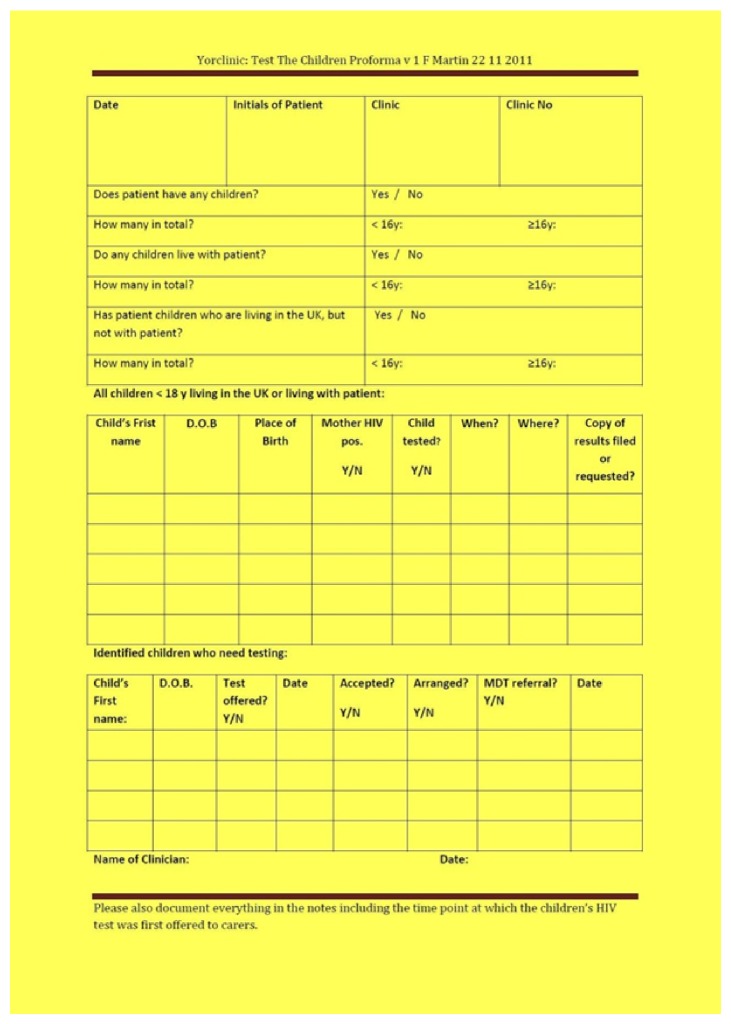
The yellow proforma (YP).

Finally we introduced a colour-coded single page proforma, the Yellow Proforma (Figure 1), which was attached to the front of all HIV patient notes for completion at their next clinic visit. This proforma was filed in the front of the notes once completed. This strategy was implemented in December 2011 and audited retrospectively in December 2012. Patients were excluded from the audit process if they were <14 years old, new patients yet to be seen, or patients who had transferred care out of our service before 2011. Duplicates were removed. Parents with children <18 years old were followed up actively, while those with older children were offered HIV testing for their children but less actively so.

The effectiveness of this audit was measured by.

1. Proportion of patients with completed proforma within 12 months,
2. Number of tested and untested children identified within 12 months,
3. Number of clinically well but untested children tested within 12 months,
4. Number of potentially unwell but untested children tested within 7 days.

## 3. Results

**Figure 2:**
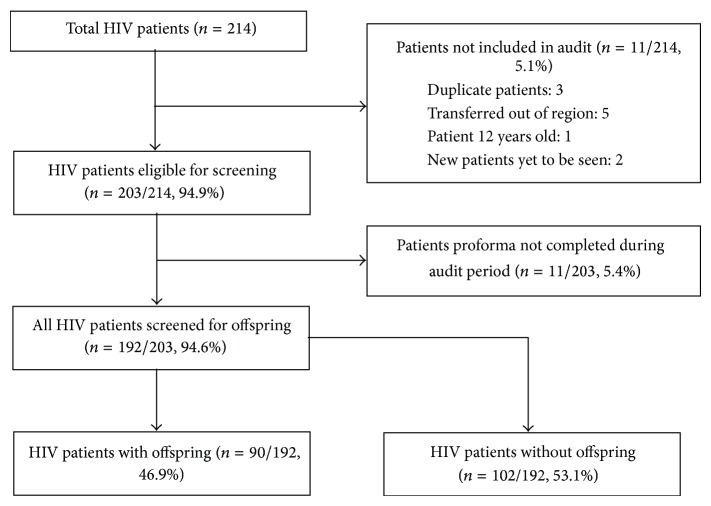
HIV patients screened for offspring. A total of 214 HIV patients attend the York/Harrogate HIV services. Eleven (5.1%) were deemed ineligible for inclusion in the audit, leaving 203/214 (94.9%) patients eligible for proforma completion. Of these, 192/203 (94.6%) had a completed proforma. A total of 90/192 (46.9%) had offspring and 102/192 (53.1%) did not.

A total of 214 HIV patients attended our services; 203 patients were eligible for inclusion in the audit. The process and outcomes of screening these patients are depicted in Figure 2.  Table 1 shows the demographics of the 203 patients. By 12 months, 192 (94.6%) patients had completed proformas. Reasons for non-completion of the remaining 11 were patients not attending or clinicians forgetting. None of these patients were known to have children who needed testing.

**Figure 3:**
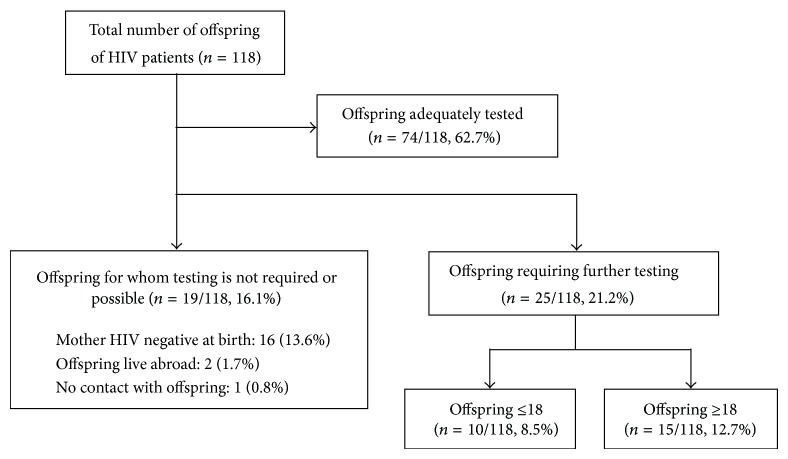
Breakdown of offspring of HIV patients. There are a total of 118 offspring from the 90 adult HIV patients identified to have offspring. Of these, 74/118 (62.7%) were adequately tested; 19/118 (16.1%) did not require testing or were unable to be tested; and 25/118 (21.2%) were in need of an HIV test. Of these, 10/118 (8.5%) were <18 years of age and were actively pursued for testing. The remaining 15/118 (12.7%) over 18 were encouraged to be tested, but less actively so.

Using the yellow proforma (YP) helped identify a total of 90 (46.9%) HIV patients with children, with a total of 118 offspring. The breakdown of these offspring and their testing status is shown in Figure 3. Of the total, 74/118 (62.7%) offspring had an adequate proof of testing available. The remaining 44/118 (37.3%) had never been tested: 19/118 (16.1%) did not require or could not be tested, leaving 25/118 (21.9%) offspring who needed further testing. All were clinically well at the time of identification. 10/118 (8.5%) offspring were ≤18 years old, and their parents were entered into the testing pathway. The median age of these children was 13.5 years (1–18), and 9/10 were of Black African origin. Their parents had been attending the local services for a median of 3.5 years (range 0–15), and all 10 children were tested HIV negative. Seven (70%) had been tested by the time of completion of the audit.

The parents of three offspring were not happy to test their child for HIV. One mother, with a history of depression, stopped attending HIV services and would send her partner to pick up her medication in order to avoid discussing HIV testing of her child. She was convinced that she had an HIV test during her antenatal care. But further investigation revealed that the opt-out testing for HIV had not been fully implemented during her pregnancy. Fourteen months after implementation of the YP, she was persuaded to have her child tested by informing her that a failure to attend would result in an automatic referral to the social services. The child was tested HIV negative by the paediatricians, and the mother started engaging again with her own HIV care.

Originating from overseas, the parents of the remaining two clinically well, but HIV untested, children reasoned that testing them was culturally unacceptable. After implementation of the YP, they promised to test the children but continued to find reasons for not doing so. Eighteen months after implementation of the YP, they were informed that their family would be referred to social services if they did not attend the appointment made with the paediatrician for HIV testing. Within 18 months of identification, their children were tested HIV negative.

The parents of the children considered to be adults were strongly encouraged to discuss HIV testing with their adult children. The same testing options, bar testing through paediatrician, were offered to all families.

## 4. Discussion

Prior to the implementation of this new strategy and despite the best intentions of the clinicians, children requiring HIV testing were being missed, since parents were approached on an ad hoc basis. Not all children were identified and follow-up of identified children was erratic and poorly documented.

We have shown that our three-pronged approach made easy, fast, and methodical identification and testing of children of HIV-positive patients, both retrospectively and prospectively, possible.

The YP reminded the clinicians to address this emotionally loaded subject and made documentation and followup easy and reliable. Using the same colour coded tool for all patients proved to patients that all patients were treated equitably and that they had not been singled out as “infectious parents.”

The paediatricians and HIV team have universally accepted the usage of YP and coordination with the new MDT at our unit. Eighteen months onward, none of the MDTs have been cancelled. Progress with each untested child was monitored and reported at each MDT until testing was achieved. The combination of the YP tool, MDT approach, the multiple HIV testing options, and the clear social service referral pathway has empowered clinicians to actively and transparently follow up the HIV testing of children living with HIV-positive patients and make appropriate referrals. As a result, clinicians feel supported by this network.

By identifying parents who find testing difficult we could focus time and resources on those particularly vulnerable children and parents, who may have otherwise slipped through the net and remained untested. Although three children had not been tested within the CHIVA recommended time frame of twelve months from identification, the novel strategy allowed identification and active management of these three cases by the MDT. None of the children were unwell, allowing us some grace time to persuade the parents eventually without deterring them from attending our services or jeopardising the children's health.

The cost and benefit of a new strategy, which led to HIV testing of all untested children, are difficult to calculate, and the implementation of this new strategy was not formally costed. However it did not seem to require more funds or time than usually spent on other auditable standards of care recommended by BHIVA, and the process facilitated appropriate use of resources on the children who needed testing. All MDT members have been very enthusiastic and proactive participants in the three monthly meetings, which last no more than one hour. The testing process utilised testing modalities were already in place independently but were unified by a single referral process.

In summary, 94.5% of HIV patients had a YP completed within 12 months. Seven out of 10 identified children needing an HIV test had been tested within 12 months of identification by the YP and one child by 14 months and two children of one family by 18 months. All children were tested HIV negative and no family needed to be referred to social services.

Despite our active efforts to test adult children, 13/15 adult children born to HIV-positive parents remain untested but could be HIV-positive. We could not find guidelines on testing and followup of this potentially infected cohort. Clear guidelines as to how to offer testing to the adult children without breaching our duty to maintain confidentiality towards our patients need to be developed urgently.

Universal implementation of our strategy might aid identification of untested children of HIV-positive patients and subsequently provide appropriate testing in a structured and timely fashion, in accordance with CHIVA and BHIVA guidance. A paper or electronic version of this method can also be used to aid data collection for a national children HIV testing audit.

## Figures and Tables

**Table 1:** Demographics of HIV-positive adults included in this audit (*n* = 203).

Median age (range)	43 (19–76)
Gender (male : female)	146 : 57
Ethnicity	
White British	140
Black African	48
Other	11
Not disclosed	4
Risk factor for infection	
Heterosexual	101
MSM	63
Bisexual	13
Other	3
Unknown	23
Median length of time attending local HIV services (range)	4 years (0–23)

## References

[1] UNAIDS report on the Global AIDS epidemic.

[2] HIV in the United Kingdom: 2012 report.

[3] Eisenhut M., Sharma V., Kawsar M., Balachandran T. (2008). Knowledge of their children's HIV status in HIV-positive mothers attending a genitourinary medicine clinic in the UK. *HIV Medicine*.

[4] Judd A., Ferrand R. A., Jungmann E. (2009). Vertically acquired HIV diagnosed in adolescence and early adulthood in the United Kingdom and Ireland: findings from national surveillance. *HIV Medicine*.

[5] Judd A., Doerholt K., Tookey P. A. (2007). Morbidity, mortality, and response to treatment by children in the United Kingdom and Ireland with perinatally acquired HIV infection during 1996–2006: planning for teenage and adult care. *Clinical Infectious Diseases*.

[6] Prime K., Judd A., Tookey P. Late diagnosis of perinatally acquired HIV infection.

[7] Richardson M. P., Sharland M. (1998). Late diagnosis of paediatric HIV infection in south west London. *British Medical Journal*.

[8] (2009). Don’t forget the children. *CHIVA & BHIVA*.

[9] HIV testing guidelines for children of HIV positive parents or siblings in the UK and Ireland.

